# Recurrent fever of unknown origin and unexplained bacteremia in a patient with a novel 4.5 Mb microdeletion in Xp11.23-p11.22

**DOI:** 10.1038/s41598-024-65341-5

**Published:** 2024-08-01

**Authors:** Cho-Rong Lee, Man Jin Kim, Sang-Heon Park, Sheehyun Kim, Soo Yeon Kim, Seong-Joon Koh, Seungbok Lee, Murim Choi, Jong Hee Chae, Sung-Gyoo Park, Jangsup Moon

**Affiliations:** 1 College of Pharmacy, Institute of Pharmaceutical Sciences, Seoul National University, 1 Gwanak-ro, Gwanak-gu, Seoul, 08826 Republic of Korea; 2 Department of Genomic Medicine, Seoul National University Hospital, 101 Daehak-ro, Jongno-gu, Seoul, 03080 Republic of Korea; 3 Department of Laboratory Medicine, Seoul National University Hospital, Seoul, 03080 Republic of Korea; 4 Department of Pediatrics, Seoul National University College of Medicine, Seoul, 03080 Republic of Korea; 5 Department of Internal Medicine and Liver Research Institute, Seoul National University College of Medicine, Seoul, 03080 Republic of Korea; 6 Department of Biomedical Sciences, Seoul National University College of Medicine, Seoul, 03080 Republic of Korea; 7 Department of Neurology, Seoul National University College of Medicine, Seoul, 03080 Republic of Korea

**Keywords:** Immunological disorders, Genetics, Immunology

## Abstract

Fever of unknown origin (FUO) remains a formidable diagnostic challenge in the field of medicine. Numerous studies suggest an association between FUO and genetic factors, including chromosomal abnormalities. Here, we report a female patient with a 4.5 Mb Xp microdeletion, who presented with recurrent FUO, bacteremia, colitis, and hematochezia. To elucidate the underlying pathogenic mechanism, we employed a comprehensive approach involving single cell RNA sequencing, T cell receptor sequencing, and flow cytometry to evaluate CD4 T cells. Analysis of peripheral blood mononuclear cells revealed augmented Th1, Th2, and Th17 cell populations, and elevated levels of proinflammatory cytokines in serum. Notably, the patient exhibited impaired Treg cell function, possibly related to deletion of genes encoding *FOPX3* and *WAS*. Single cell analysis revealed specific expansion of cytotoxic CD4 T lymphocytes, characterized by upregulation of various signature genes associated with cytotoxicity. Moreover, interferon-stimulated genes were upregulated in the CD4 T effector memory cluster. Further genetic analysis confirmed maternal inheritance of the Xp microdeletion. The patient and her mother exhibited X chromosome-skewed inactivation, a potential protective mechanism against extensive X chromosome deletions; however, the mother exhibited complete skewing and the patient exhibited incomplete skewing (85:15), which may have contributed to emergence of immunological symptoms. In summary, this case report describes an exceptional instance of FUO stemming from an incompletely inactivated X chromosome microdeletion, thereby increasing our understanding of the genetics underpinning FUO.

## Introduction

Fever of unknown origin (FUO) is characterized by persistent or multiple febrile episodes with no obvious cause, despite comprehensive evaluation (i.e., routine blood tests, autoimmune screening, blood cultures, inflammatory marker assessment, chest radiography, and abdominal imaging)^1^. Traditionally, FUO is categorized as infectious or non-infectious; however, the incidence of infectious etiology has decreased in recent times, and is now superseded by autoimmune or autoinflammatory conditions. Many causes may have an underlying genetic basis; indeed, advances in genetic diagnostics have led to the finding that autoinflammatory and autoimmune diseases result from genetic factors. Molecular diagnosis not only increases our understanding of infection risks and non-infectious comorbidities, but also facilitates more precise treatment strategies. The International Union of Immunologic Societies (IUIS) Committee, recognizing the rapid expansion of diagnosable primary immunodeficiency diseases (PIDs), released an interim update in 2021, encompassing 26 new monogenic gene defects associated with inborn errors in immunity (IEI)^2^. The rise of high-throughput DNA sequencing has accelerated discovery of genes linked to monogenic IEI, leading to the International Union of Immunological Sciences Expert Committee to change terminology from ‘primary immunodeficiency’ to ‘IEI’; this change reflects the fact that these conditions have more complex etiologies than simple susceptibility to infection. IEIs encompass a spectrum of immune dysregulation, including autoimmunity, autoinflammation, cancer susceptibility, and bone marrow failure, thereby underscoring the complex interplay between infection control and autoimmunity. Here, we share a unique case of suspected monogenic IEI^3^.

One notable example of IEIs is Immunodysregulation Polyendocrinopathy Enteropathy X-linked (IPEX) syndrome, primarily caused by functional FOXP3 deficiency. Typical IPEX syndrome manifests with severe autoimmune symptoms such as enteropathy, dermatitis, thyroiditis, and type 1 diabetes, often leading to fatality within the first 2 years of life. Moreover, there have been documented cases of atypical IPEX presentations associated with some mutations in *FOXP3*^4,5^. In the previous report which reports the cases of *FOXP3* genetic deletion (Xp11.23), healthy carriers showed skewed exclusive expression of wild-type *FOXP3* allele*,* signifying protective skewing of X chromosome inactivation (XCI)^6^.

Here, we describe a female patient heterozygous for previously unreported Xp11.23-Xp11.22 4.5 Mb microdeletions, including *FOXP3* and *WAS*. In our case, microdeletion of Xp11.23-22 resulted in an immunologically defective phenotype. In general, X-linked disorders only affect males; however, this case was a 22-year-old Korean female who suffered from recurrent bacteremia. She presented with persistent fever and colitis, and was treated with several immunosuppressants, none of which led to substantial improvement. To the best of our knowledge, this is the first report of this specific Xp11.23-p11.22 microdeletion in a female patient presenting with immunological defects.

## Results

### Identification of an X chromosome microdeletion in a patient with recurrent fever and bacteremia

**Figure 1:**
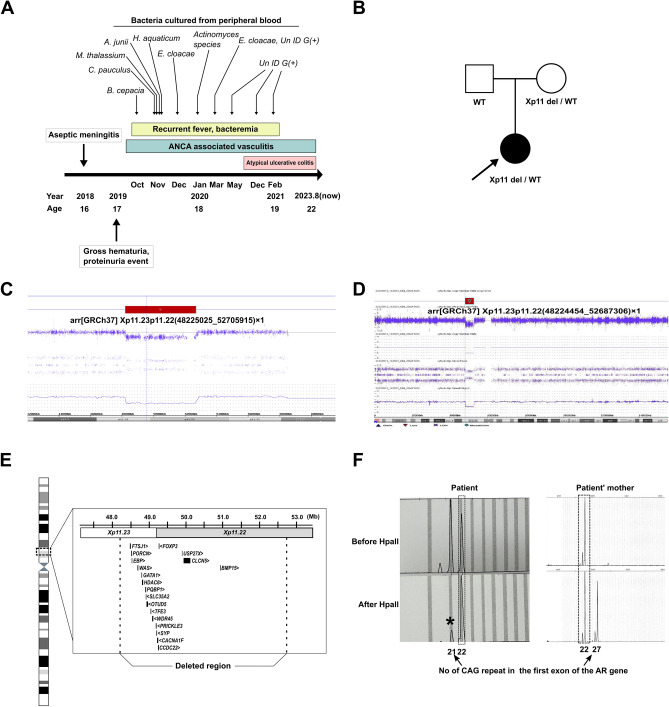
Clinical presentation and genetic findings. (**A**) A time line illustrating the patient’s medical history is presented. (**B**) Pedigree of the patient. Chromosomal microarray detected a deletion of approximately 4480 kb on the X chromosome (**C,D**), which included 19 genes reported in Online Mendelian Inheritance in Man (OMIM, https://www.omim.org/) (**E**). (**F**) A skewed X chromosome inactivation pattern, with a ratio greater than 85:15, was observed, indicating inactivation of the 22repeat allele. It is noteworthy that the patient exhibited approximately 85% skewing (indicated by an asterisk), whereas the mother displayed 100% skewing.

The patient had an unremarkable medical history, with the exception of atopic dermatitis and asthma during childhood, both of which improved with age. In 2018, the patient experienced an episode of aseptic meningitis. In 2019, multiple events of gross hematuria and proteinuria occurred, followed by recurrent fever up to over 40 °C and confirmation of bacteremia (Fig. 1A) caused by atypical bacteria usually not found in a clinical setting. In addition, CT scans revealed waxing and waning ground-glass opacities (GGO) in both lungs. We also found renal involvement (hematuria and proteinuria), a history of asthma, and positivity for antineutrophil cytoplasmic antibodies (p-ANCA) to MPO, raising a suspicion of ANCA-associated vasculitis. Subsequently, in the second half of 2020, the patient suffered from persistent diarrhea and hematochezia, and atypical ulcerative colitis was suspected with chronic colitis diagnosed by colonoscopy. Meanwhile, there was no significant family history (Fig. 1B).

To exclude a genetic cause underlying these clinical manifestations, we performed trio whole-exome sequencing (WES) (Supplementary Table S1). However, no clinically significant sequence variants were detected via trio WES. Additionally, CCR-CNV, an in-house CNV prediction tool, indicated a heterozygous deletion of the X chromosome (data not shown)^7^. Inspection of the region by integrative genomics viewer (IGV) revealed reduced depth (Supplementary Fig. S1). To confirm this deletion, we conducted chromosomal microarray analysis (CMA), which revealed a deletion of approximately 4,480 kb on the X chromosome (arr[GRCh37] Xp11.23p11.22(48225025_52705915) × 1; Fig. 1C). We also conducted whole-genome sequencing on the patient, and we could not identify any additional pathogenic variants apart from the Xp11.23-p11.22 microdeletion. This deletion was also identified in the patient’s mother (Fig. 1D), yet she shows no obvious symptoms or signs. This region encompasses 19 genes reported in Online Mendelian Inheritance in Man (OMIM, https://www.omim.org/), including *FOXP3* and *WAS* (Fig. 1E). Considering that this was a heterozygous X chromosome deletion in a female patient, we performed an XCI assay to assess skewing, and found a skewed XCI pattern in both the patient and her mother (Fig. 1F), indicating inactivation of the 22-repeat allele in the patient (> 85:15). Notably, the patient exhibited incomplete skewing (approximately 85%), whereas the mother showed complete skewing (100%). To the best of our knowledge, this is the first report of a female patient presenting with a Xp11.23-p11.22 microdeletion with a skewed XCI pattern, in which the symptomatic mutated alleles are activated preferentially.

### Immunological features of the patient

**Figure 2:**
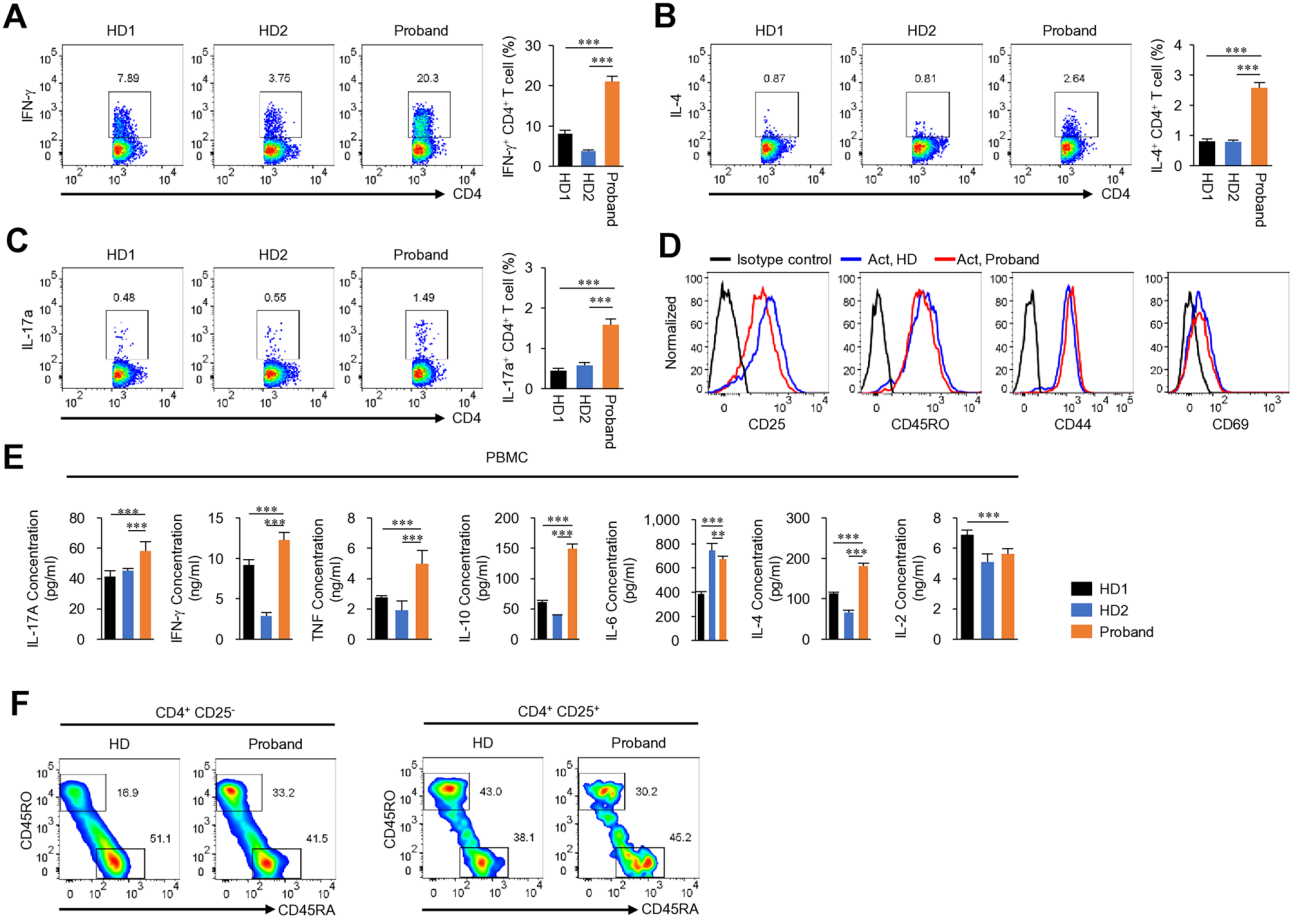
Immune profile of the patient with an Xp11.23-11.22 microdeletion. Flow cytometry analysis of freshly isolated PBMCs was performed. Cells were gated on the CD4 population to analysis Th1 (**A**), Th2 (**B**), and Th17 (**C**) populations. PBMC were stimulated with PMA/ionomycin in the presence of Brefeldin A for 5 h. (**D**) The histograms show expression of activation markers (CD25, CD44, and CD69) and memory markers (CD45RO) by CD4 cells stimulated with anti-CD3 and anti-CD28 antibodies. (**E**) Cytokine levels in serum. (**F**) Naïve and memory phenotype analysis in CD4 T cells or Treg cells from PBMC by flow cytometry. *HD* healthy donor. In experiments (**A–C,E**), the samples were tested in triplicate. Results are expressed as the mean ± SD. **p < 0.01, ***p < 0.001 by one-way ANOVA and Tukey post-test (**A–C,E**).

**Figure 3:**
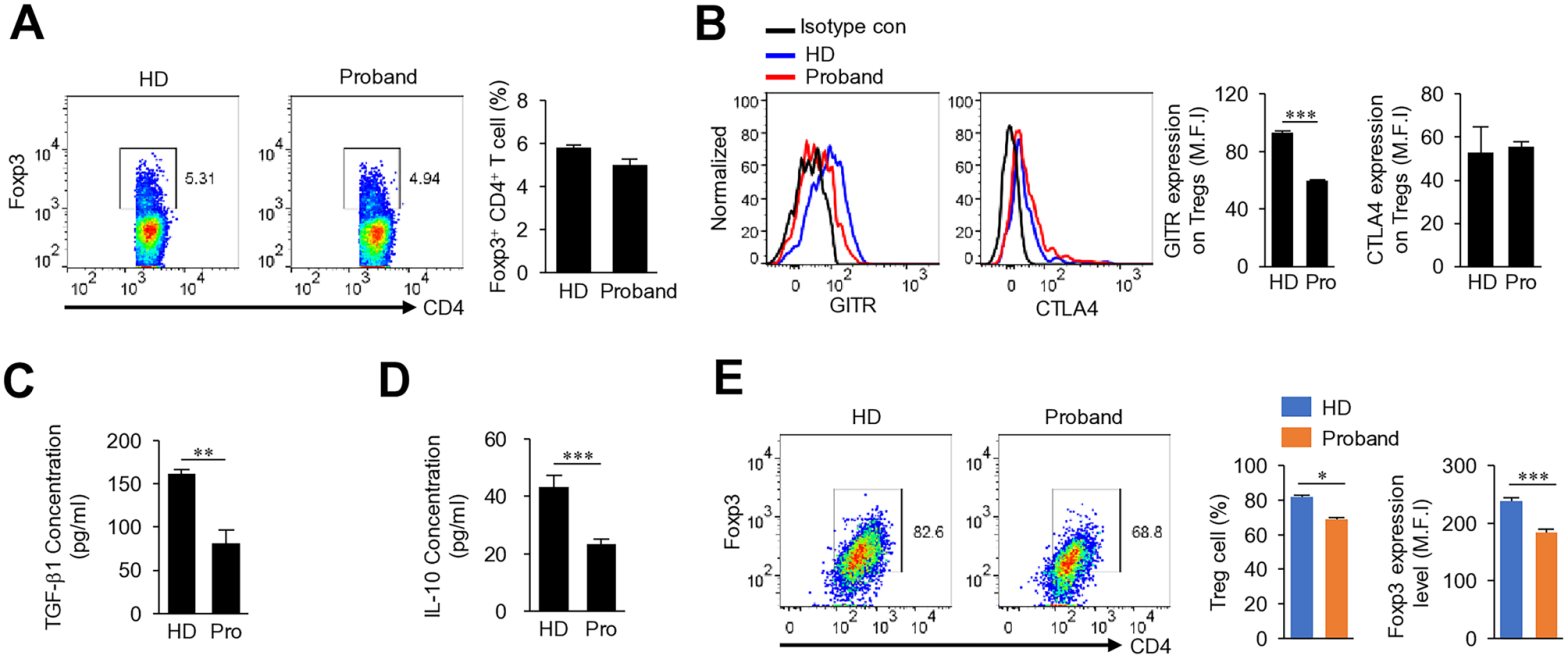
Treg cell profiles of the patient with an Xp11.23-11.22 microdeletion. (**A**) Flow cytometry analysis of Treg cells. (**B**) Flow cytometry analysis of GITR or CTLA-4 expression levels in Treg cells from PBMC. (**C,D**) Cytokine expression by Treg cells. (**E**) Flow cytometry analysis of in vitro-induced Treg cells (iTregs). iTreg cells were generated from CD4 T cells by stimulation with anti-CD3 and anti-CD28 antibodies in the presence of TGF-β. *MFI* mean fluorescence intensity, *HD* healthy donor. In experiments (**B–E**), the samples were tested in triplicate. Results are expressed as the mean ± SD. *p < 0.05, **p < 0.01, ***p < 0.001 by Student’s t-test (**B–E**—right graph) or Mann–Whitney *U* test (**A,E**—left graph).

In order to determine whether the defect of the immune cells of patient lead to phenotype, we applied immune cell analysis from patient with Xp microdeletion. Analysis of the patient’s peripheral blood mononuclear cells (PBMCs) revealed a significant increase in the Th1, Th2, and Th17 cell populations (Fig. 2A–C). Surface expression of activation markers such as CD25, CD44, and CD69, as well as the memory marker (CD45RO), did not differ between patient and healthy donor (HD) CD4 T cells after in vitro stimulation (Fig. 2D). Serum cytokines, including IL-17A, IFN-γ, TNF, IL-10, IL-6, IL-4, and IL-2 levels from the patient and HD were measured using a Cytokine Bead Array. The results revealed an increase in proinflammatory cytokine levels in the patient’s serum (Fig. 2E). In addition, the effector/memory CD4 T cell population was higher in the patient than in the HD (Fig. 2F). Our case is heterozygous for the deleted Xp11.23-p11.22 chromosome region, but various genes including *FOXP3* and *WAS* genes, which are important for Treg cell function^8,9^. It is somewhat similar to, but different from, previously reported cases of *WAS* and *FOXP3* deficiency associated with Xp11.23^9,10^. The proportion of Treg cells in the patient was no different from that in the HD (Fig. 3A), while the level of GITR was lower than that in the HD (Fig. 3B). Remarkably, there was a notable reduction in the levels of the Treg cell function-related cytokines TGF-β1 and IL-10 levels in the patient (Fig. 3C,D). In addition, induction of in vitro-induced Treg (iTreg) cell differentiation was less efficient in the patient than in the HD (Fig. 3E). The observed iTreg differentiation differences in the same type of cells under in vitro conditions are presumed to be due to an intrinsic predisposition driven by the combined effect of Xp microdeletion and skewed inactivation within CD4 T cells. These dysfunctional Treg cells may render the patient susceptible to the development of immune-mediated inflammatory diseases.

### scRNA-Seq of CD4 T cells from the patient and HD

**Figure 4:**
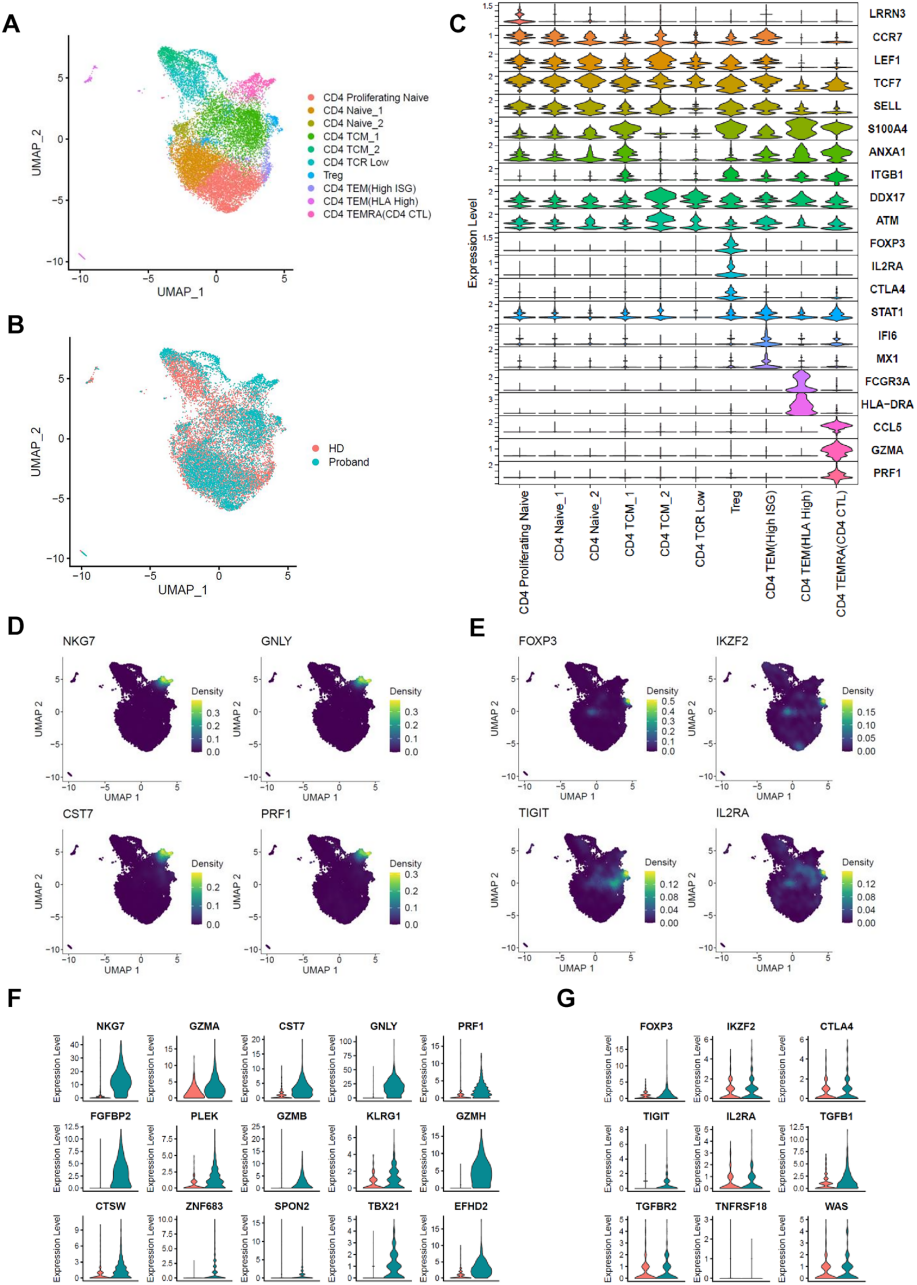
Single cell RNA-seq analysis of CD4 T cells from a healthy donor and from the patient with an Xp 11.23-11.22 microdeletion. scRNA-seq was performed on CD4 T cells from the patient and healthy donor. (**A**) UMAP embedding of 17,093 CD4 T cells presenting as 10 different clusters. (**B**) UMAP embedding of merged scRNA-seq profiles from CD4 T cells, including 9886 and 7207 cells isolated from PBMCs of a healthy donor and the proband, respectively. (**C**) Violin plots showing expression patterns of selected canonical cell markers within the 10 clusters. (**D,E**) Density plot produced by the Nebulosa package showing expression of CD4 CTL-related genes (*NKG7, GNLY, CST7, PRF1*) (**D**) and Treg cell-related genes (*FOXP3, IKZF2, TIGIT, IL2RA*) (**E**). (**F,G**) Violin plots showing expression of CD4 CTL signature genes (**F**) and Treg cell signature genes (**G**) between HD and the Proband.

Given the importance of CD4 T cells for disease pathogenesis, we next sequenced CD4 T cell RNA using the 10× Genomics Gem Code Chromium platform to explore transcriptomic differences between the patient and HD. After quality control, we obtained expression profiles for 9886 and 7207 CD4 T cells from the HD and patient, respectively. Dimension reduction by Uniform Manifold Approximation and Projection (UMAP) identified 10 clusters: three clusters of CD4 naïve T cells and seven clusters of CD4 effector/memory T cells, each with unique signature genes (Fig. 4A). To identify the specific CD4 T cell types associated with symptoms, we integrated data from the HD and patient (Fig. 4B). We next identified differentially expressed RNA markers in each cluster, and found that each exhibited distinct molecular patterns and biomarkers (Fig. 4C, Supplementary Fig. S2).

To identify differences in cell composition across samples, the clusters were examined separately (Supplementary Fig. S3A), and the percentage of cells within the 10 major cell clusters was calculated for each individual (Supplementary Fig. S3B). Most cell types exhibited overlap between the patient and HD. Notably, the patient had a much higher percentage of cytotoxic CD4 T cells (CD4 CTLs) than the HD (Supplementary Fig. S3B). In general, cytotoxic CD4 T cell (CD4 CTL) numbers are low under normal conditions^11,12^. In humans, CD4 CTL numbers increase in response to chronic viral infection^13–15^, anti-tumor responses^16,17^ and several autoimmune diseases^18–20^. The CD4 CTL cluster was characterized by high expression of genes associated with the cytotoxic functions of CD8^+^ T cells and natural killer (NK) cells; these included *NKG7, GZMA, CST7, GNLY, PRF1, FGFBP2, PLEK, GZMB, KLRG1, GZMH, CTSW*, *ZNF683, SPON2, TBX21*, and *EFHD2*. These CD4 CTL signature genes were overall higher in cluster of the patient than healthy donor (Fig. 4D,F). Although expression of Treg cell function-related proteins was reduced (Fig. 3), scRNA-seq did not reveal a change in expression of Treg-related genes (Fig. 4E,G). Furthermore, the proportion of CD4 TEMs was considerably lower in the patient than in the HD (Supplementary Fig. S3B), while expression of interferon (IFN)-stimulated genes (ISGs) including *IFI44L, ISG15, MX1, IRF7*, and *IRF1* was upregulated in the CD4 TEM (High ISG) cluster from the patient (Supplementary Fig. S3C,D). These findings suggest that immunological abnormalities in the patient from changes in gene expression of CD4 T cells induced by Xp microdeletion.

### Marked clonal expansion of CD4 CTLs from the patient

**Figure 5:**
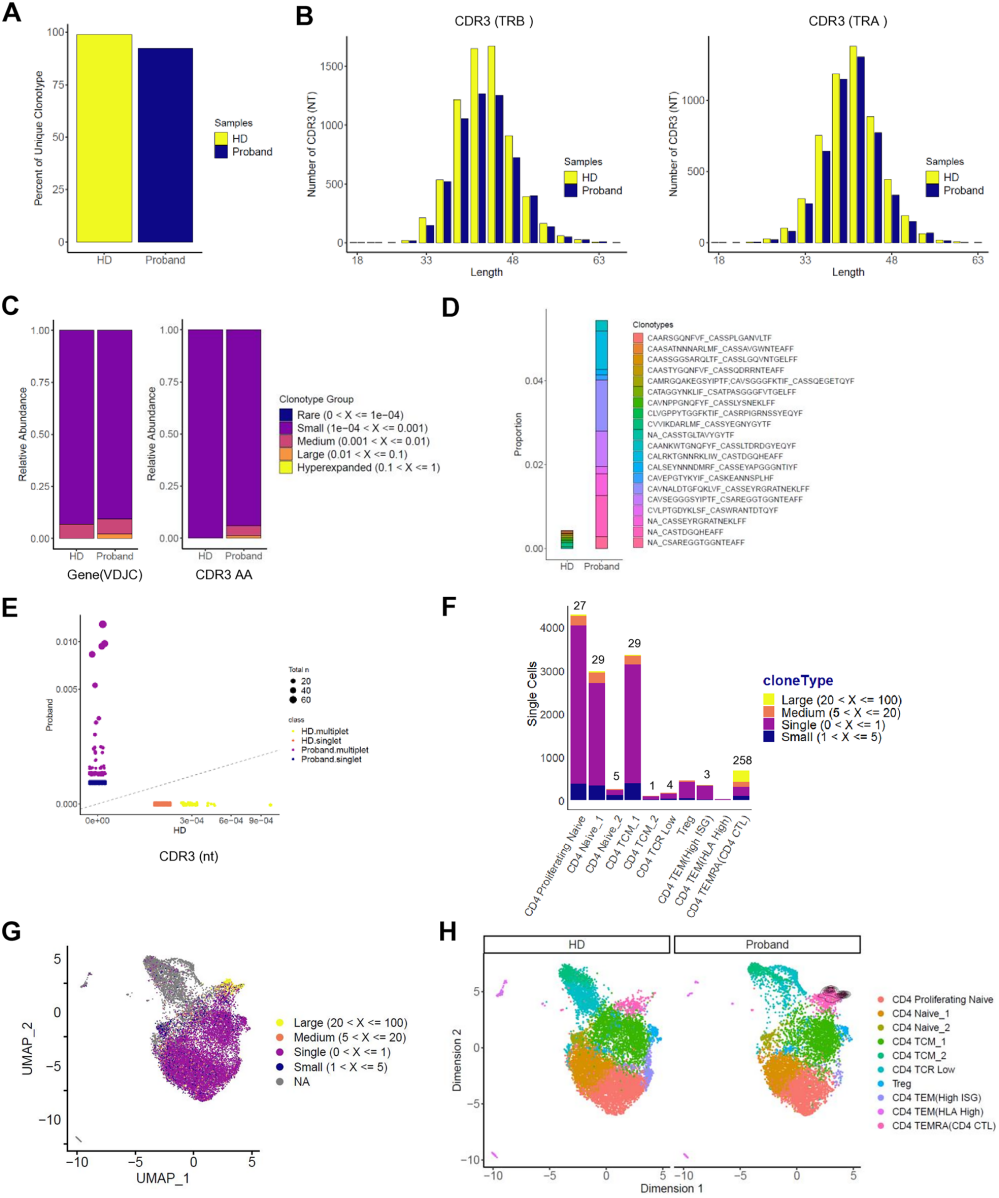
Marked clonal expansion of the patient’s CD4 CTLs. Single cell TCR clonotype analysis using the 10× Genomics platform. Data were analyzed with Cell Ranger VDJ Scripts and plotted using scRepertoire R package. (**A**) Scaled unique clonotypes by total number of TCRs sequenced by sample (HD, Proband). (**B**) Analysis of CDR3 nucleotide length in TCRs (TRB: β chain, TRA: α chain) from each sample. (**C**) Clonal homeostatic space representation (clonal space occupied by clonotypes of specific proportions) of TCRs across samples. (**D**) The 10 most expanded TCR clones. Amino acid sequences represent TCR CDR3 regions expressed as “TCRα_TCRβ”. (**E**) Scatter plot of CDR3 nucleotide usage comparing the two samples. Circle size indicates total abundance of each expanded clonotype. (**F**) Cell counts by cluster assigned into large frequency ranges. (**G**) UMAP visualization of clonal expansion detected in CD4 T cells. (**H**) Distribution of cells over the reference map for the expanded clonotypes by sample.

To further determine the characteristics of CD4 T cells in the patient, TCR sequencing was performed using the 10× Genomics V(D)J-enriched library. CD4 T cell TCR diversity was assessed in the patient and HD. Definition of clonotypes was based on CDR3 sequencing of both TCR alpha and beta chains using the Cell Ranger analysis pipeline. Clonotypic analysis of the TCR was conducted using the scRepertoire package^21^. We calculated the percentage of each unique T cell clonotype; most CD4 T cells from the HD contained unique TCRs, while those from the patient showed a reduced percentage of unique clonotypes (Fig. 5A). Next, we analyzed the length of the CDR3 nucleotides in the TCR alpha and beta chains. The distribution of CDR3 length in both TRA and TRB from the HD and patient were similar, but not completely identical, to the normal distribution. More clonotypes were found at 33, 36, 39, 42, 45, 48 nucleotides from healthy donor (Fig. 5B). These findings also indicate that the TCR repertoire of patient is less diverse than that of the HD. Moreover, the relative abundance of highly expanded clonotypes was higher in the patient (Fig. 5C). From the TCR repertoire analysis of these paired TCRαβ sequences, we represented the top 10 TCR clonotypes for each sample. In the case of HD, there were few overlapping clones in the top 10; however, the proportion of expanded clones in the top 10 was higher in the patient (Fig. 5D). No TCR clonotype was shared between the patient and HD (Fig. 5E). Most of these expanded TCR clonotypes were found in the CD4 TEMRA cluster (CD4 CTL) (Fig. 5F–H). In the CD4 TEMRA cluster (CD4 CTL), in which expanded clones comprised the highest proportion, there were a total of 258 large clone types, ranging from 20 to 100; of these, 256 expanded clones were found in the patient (Fig. 5F). Public databases were mined to assess the known antigen specificity of CDR3 sequences. We analyzed the top five expanded clonotypes in CD4 CTLs using TCR databases VDJdb^22^ and McPAS-TCR^23^; however, none of the clonotypes were found. The CD4 CTLs are known to be highly heterogenous across patients^24^; therefore, it is not entirely surprising that the expanded clonotypes in the patient did not match those deposited in TCR databases.

## Discussion

Here, we performed a comprehensive immune cell analysis to characterize a patient with an X chromosome microdeletion. We noted marked expansion of the CD4 CTL cell population associated with cytotoxic functions, coupled with a decrease in Treg cell function. These findings raise the possibility that CD4 T cells play different pathological roles in this patient. However, there are several limitations that should be borne in mind when interpreting these data. It is unclear what drives changes in TCR-mediated clonal expansion of CD4 CTL cells. Although CD4 CTLs play important roles in various autoimmune diseases, including ulcerative colitis and disease pathogenesis^12,25^, little is known about the mechanism by which they are generated, their heterogeneity, or their therapeutic targets in humans. Despite these limitations, this case is unique in that the patient is female; most X-linked recessive diseases show a marked male predominance.

Most mutations on the X chromosome primarily affect males; this is because a mutation in a single copy of any gene on the X chromosome leads to disease manifestation. However, the phenomenon of skewed XCI can buck this trend, potentially causing disease in females who carry two X chromosomes^26–28^. The incomplete skewing deviation observed in the patient indicates a potential link between the expression of the deleted mutant allele within the X chromosome region and the differentiation of iTreg cells in naïve CD4 T cells, also possibly influencing Treg cell function.

In our case, classical symptoms associated with *FOXP3* deficiency were notably absent. Instead, the patient predominantly exhibited recurrent fever and bacteremia. One plausible hypothesis for this unusual presentation is that *FOXP3* deficiency may have impaired the function of intestinal Treg cells, leading to reduced levels of IL-10. This, in turn, could result in persistent intestinal inflammation, ultimately causing colitis. Consequently, colitis-related complications such as intestinal blood vessel rupture and gastrointestinal bleeding could occur. Simultaneously, compromised intestinal barrier function might allow gut bacteria to enter the bloodstream, leading to bacteremia and fever. Notably, studies show that intermittent depletion of Foxp3^+^ Treg cells exacerbates intestinal inflammation, emphasizing the crucial role of Foxp3^+^ Treg cells in maintaining mucosal intestinal balance and controlling inflammation^29^. Furthermore, it is worth mentioning that IL-10 knockout mice develop colitis associated with disruption of gut mucosal barrier function, resulting in increased permeability and endotoxemia^30^.

Another gene, *WAS*, which also resides within the X chromosome deletion region, might have caused symptoms in the patient. Wiskott–Aldrich syndrome (WAS) is another type of X-linked primary immunodeficiency (PID) resulting from mutation of the gene that encodes the Wiskott–Aldrich syndrome protein (WASp)^31^. WASp plays an important role in nTreg cells, and WASp deficiency can trigger autoimmune diseases due to nTreg cell dysfunction^9,32^. Our case shares some clinical features with WAS patients, including recurrent bacterial infections and vasculitis. There are several other reports of females showing clinical signs of WAS, some of which caused expression of a mutant allele through X-skewed inactivation^33–35^. Thus, skewed X-inactivation of genes within the X chromosome region is the most likely mechanism underlying the clinical symptoms of female carriers of X-linked recessive disorders.

## Conclusion

Here, we report a patient who underwent next-generation sequencing to diagnose the cause of immunodeficiency. We identified a novel microdeletion on the X chromosome. The skewed XCI led to the clinical phenotype of this female patient, who harbored an Xp11.23-11.22 microdeletion. Identification of functional defects in Treg cells, coupled with patient-specific expansion of CD4 CTLs, may lead to development of targeted treatments.

## Materials and methods

### PBMC isolation

Peripheral blood mononuclear cells (PBMCs) were isolated from the proband’s peripheral venous blood (collected from a Hickman catheter) by density gradient centrifugation on Ficoll-Paque™ PLUS (GE Healthcare Life Sciences). Isolated cells were stained with the indicated fluorochrome-conjugated antibodies and analyzed by a Canto II flow cytometer (BD Biosciences).

### Whole-exome sequencing

To understand the genetic basis of the clinical manifestations, whole-exome sequencing (WES) of the patient and her parents was performed. DNA extracted from peripheral blood was enriched using Agilent SureSelect All Exons V6 (Agilent Technologies, Santa Clara, CA, USA), followed by sequencing on a HiSeq system (Illumina, San Diego, CA, USA), with 100 bp paired-end reads. Bioinformatics analysis, from alignment to annotation, was performed by NextGene (Version 2.4.0.1; Softgenetics, State College, PA, USA).

### Chromosomal microarray analysis (CMA)

CMA utilized the CytoScan Dx Assay (Affymetrix, Santa Clara, CA, USA) following the manufacturer’s instructions. The array comprised 1.95 million copy number probes and 750,000 single nucleotide polymorphism probes. Data analysis was performed using Chromosome Analysis Suite Dx (Affymetrix), with CNV coordinates referenced to the human genome assembly GRCh37/hg19.

### X chromosome inactivation (XCI) assay

X-inactivation analysis involved PCR targeting a highly polymorphic CAG repeat within the first exon of the androgen receptor gene^36^. The PCR utilized the following primers specific for the CAG repeats: forward: 5′-FAM-TCCAGAATCTGTTCCAGAGCGTGC-3′; reverse: 5′-GCTGTGAAGGTTGCTGTTCCTCAT-3′. The PCR conditions were as follows: initial denaturation at 94 °C for 5 min, followed by 35 cycles comprising denaturation at 94 °C for 30 s, annealing at 55 °C for 60 s, extension at 72 °C for 90 s, and a final cycle at 72 °C for 5 min and 60 °C for 45 min. PCR products from both digested and undigested DNA were analyzed using an ABI PRISM 3130 Genetic Analyzer (Applied Biosystems, Foster City, CA, USA) and processed with GeneMapper Software v.3.7 (Applied Biosystems).

### Flow cytometry analysis

Isolated or differentiated cells were stained with the indicated antibodies and analyzed on a FACSCanto II (BD Biosciences) or a Guava flow cytometer (Millipore). Live cells were gated by forward and side scatter. For intracellular staining, cells were fixed and permeabilized with Foxp3 staining buffer (eBioscience). Data were analyzed by FlowJo software.

### Cytokine measurement

Serum cytokines including IL-17, IFN-γ, TNF, IL-10, IL-6, IL-4 and IL-2 in the patient’s or healthy donors’ serum were measured with the Cytokine Bead Array (BD Biosciences, San Jose, Calif) by using a Canto II flow cytometer (BD Biosciences).

### Single cell RNA-seq and data processing

PBMCs were stained with anti-human CD4 (OKT4, eBioscience) and CD4 T cells were isolated using a FACSAria III instrument (BD Biosciences). Single cell 5ʹ RNA-Seq libraries were generated using a Single Cell 5ʹ Library and Gel Bead Kit and Chromium Controller (10× Genomics) according to the manufacturer’s instructions. Single Cell VDJ, 5ʹ GEX, and feature barcode libraries were sequenced on the Illumina sequencing system with paired-end reads. After assessing the quality of the sequencing libraries, they were loaded onto an Illumina Novaseq platform. Cell Ranger (v5.0.0, from 10× Genomics) was used to align scRNA-seq reads to the human reference genome (assembly and annotation, GRCh38-2020-A, GRCh38-alts-ensembl-5.0.0) to generate gene-by-cell count matrices. All samples passed the quality control measures for Cell Ranger version 5.0.0. scRNA-seq sample data were analyzed using Seurat 4.3.0 and the parameters recommended by Seurat software.

### TCR clonotype analysis

The TCR clonotypes were identified by analyzing the variable, diversity, and joining genes of the TCR alpha and beta chains in conjunction with the nucleotide sequences of the complementarity-determining region 3 (CDR3) regions. TCR data was analyzed using the scRepertoire package.

### Statistical analysis

Statistical analysis was performed using GraphPad Prism (10.2.0). All values are presented as the mean ± standard deviation. Significant differences in the mean values between the two groups were determined using Student’s t-test or Mann–Whitney U test. For analysis of multiple datasets, one-way ANOVA was used with Tukey post-test. For all statistical analyses, p < 0.05 was considered to statistically significant.

### Ethical approval

The study was approved by the Institutional Review Board of Seoul National University Hospital (IRB No. H-2204-112-1317). All subjects provided informed consent according to ethical guidelines for academic research, and all experiments were performed in accordance with the relevant guidelines and regulations.

### Supplementary Information

Supplementary Information.

## Data Availability

The dataset generated and/or analyzed during the current study were deposited in NCBI GEO repository (http://www.ncbi.nlm.nih.gov/geo/) (GSE255381).
