# Supplementary material for: Recurrent fever of unknown origin and unexplained bacteremia in a patient with a novel 4.5 Mb microdeletion in Xp11.23-p11.22
Source: Sci Rep. 2024 Aug 1;14:17801. doi: 10.1038/s41598-024-65341-5 (PMC11294525; doi:10.1038/s41598-024-65341-5)
Supplement: Supplementary file 1 — Supplementary Information. [file 41598_2024_65341_MOESM1_ESM.docx]

**Supplementary Material**

**Recurrent Fever of Unknown Origin and Unexplained Bacteremia in a Patient with a Novel 4.5 Mb Microdeletion in Xp11.23-p11.22**

Cho-Rong Lee ^1, †^, Man Jin Kim^2, 3, †^, Sang-Heon Park ^1^, Sheehyun Kim^2^, Soo Yeon Kim^2, 4^, Seong-Joon Koh^5^, Seungbok Lee^2, 4^, Murim Choi^6^, Jong Hee Chae^2, 4^, Sung-Gyoo Park^1*^, Jangsup Moon^2, 7*^

**Author affiliations:**

^1^Institute of Pharmaceutical Sciences, College of Pharmacy, Seoul National University, Seoul 08826, Republic of Korea.

^2^Department of Genomic Medicine, Seoul National University Hospital, Seoul 03080, Republic of Korea.

^3^Department of Laboratory Medicine, Seoul National University Hospital, Seoul 03080, Republic of Korea.

^4^Department of Pediatrics, Seoul National University College of Medicine, Seoul 03080, Republic of Korea.

^5^Department of Internal Medicine and Liver Research Institute, Seoul National University College of

Medicine, Seoul 03080, Republic of Korea.

^6^Department of Biomedical Sciences, Seoul National University College of Medicine, Seoul 03080, Republic of Korea.

^7^Department of Neurology, Seoul National University Hospital, Seoul 03080, Republic of Korea.

**^†^These authors contributed equally to this work**

***Correspondence to:**

Jangsup Moon, MD, PhD

Department of Genomic Medicine and Department of Neurology, Seoul National University Hospital, 101 Daehak-ro, Jongno-gu,

Seoul 03080, Republic of Korea

Tel: +82-2-2072-4265

E-mail: [jangsup.moon@gmail.com](mailto:jangsup.moon@gmail.com)

Sung-Gyoo Park, PhD

Institute of Pharmaceutical Sciences, College of Pharmacy, Seoul National University,

1 Gwanak-ro, Gwanak-gu,

Seoul 08826, Republic of Korea

Tel: +82-2-880-8180

E-mail: riceo2@snu.ac.kr

**Supplementary material includes**

**Table S1**

**Figure S1-S3**

**Table S1. Whole exome sequencing run summary**

| **Case** | **Proband** | **Mother** | **Father** |
| --- | --- | --- | --- |
| Read length (bp) | 2 x 100 | 2 x 150 | 2 x 150 |
| Number of reads (M) | 124.4 | 70.0 | 70.4 |
| Mean coverage depth (X) | 171 | 95 | 95 |
| % of reads on target | 75.0% | 62.1% | 61.3% |
| % of bases covered at least 5x | 99.8% | 99.6% | 99.7% |
| % of bases covered at least 10x | 99.1% | 99.0% | 99.0% |

**
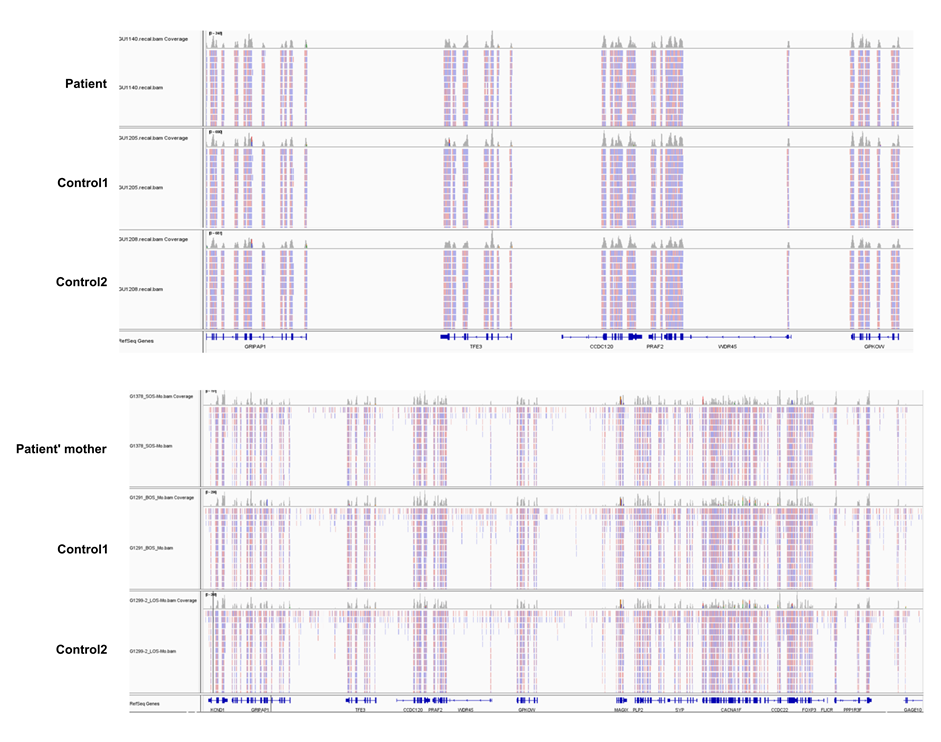
**

Supplementary Figure S1

The integrative genomics viewer (IGV) depicting the Xp11.23p11.22 region


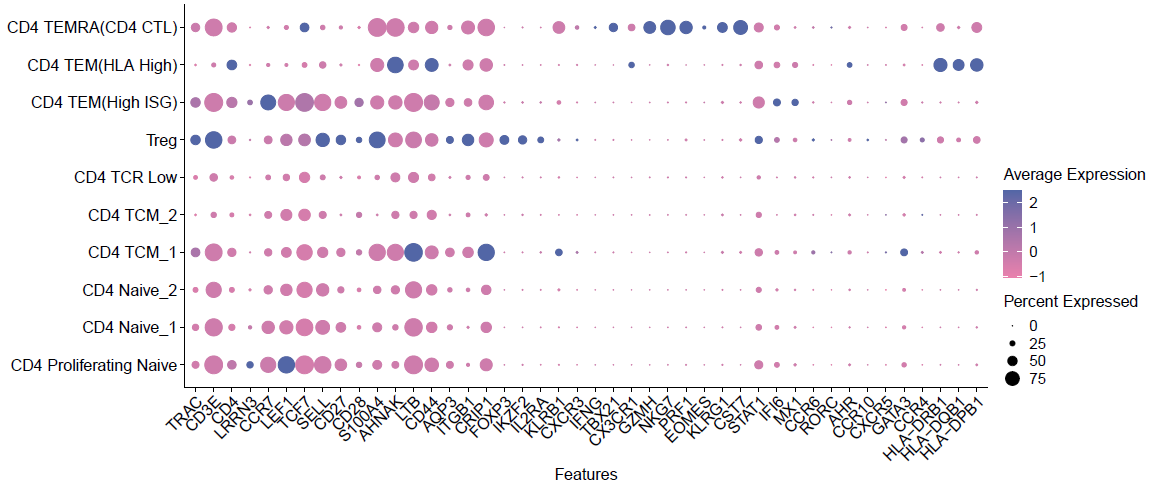

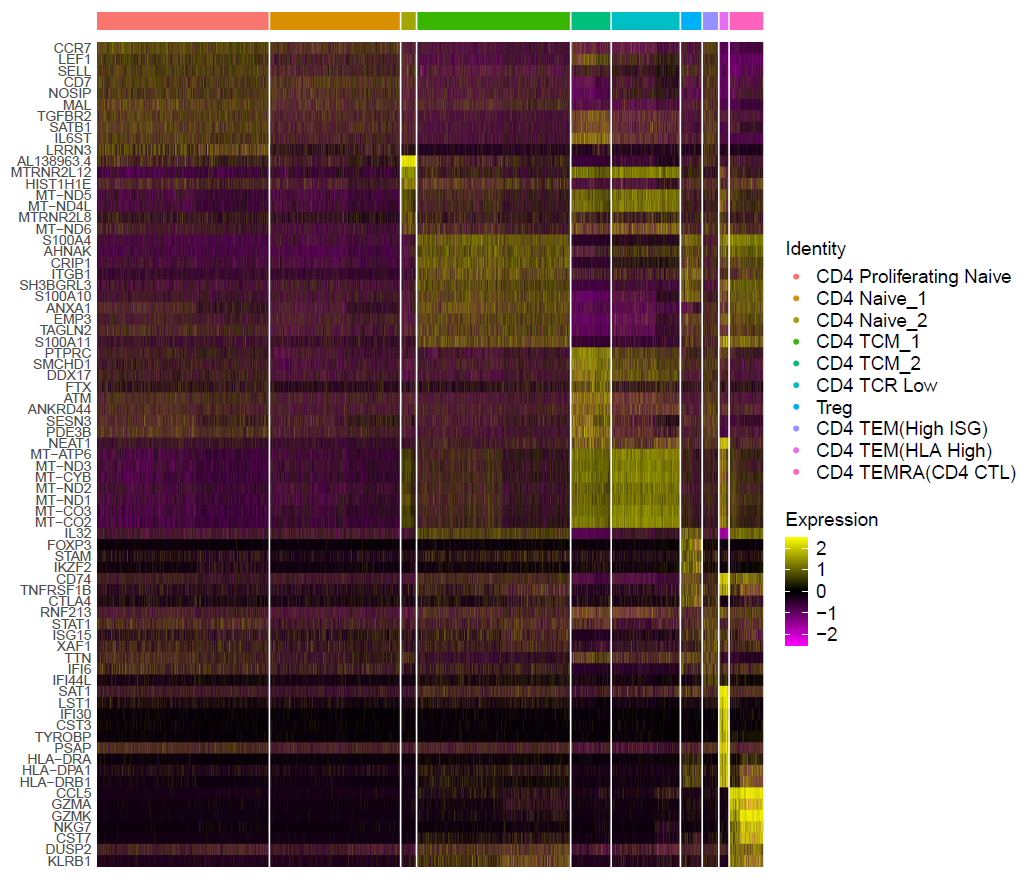


**A**

**B**

Supplementary Figure S2

(A) Heatmap representing the differentially expressed genes in identified clusters of CD4 T cells.

(B) Dot plot depicting gene expression levels and percentage of cells expressing genes associated with CD4 T cell

phenotypes.


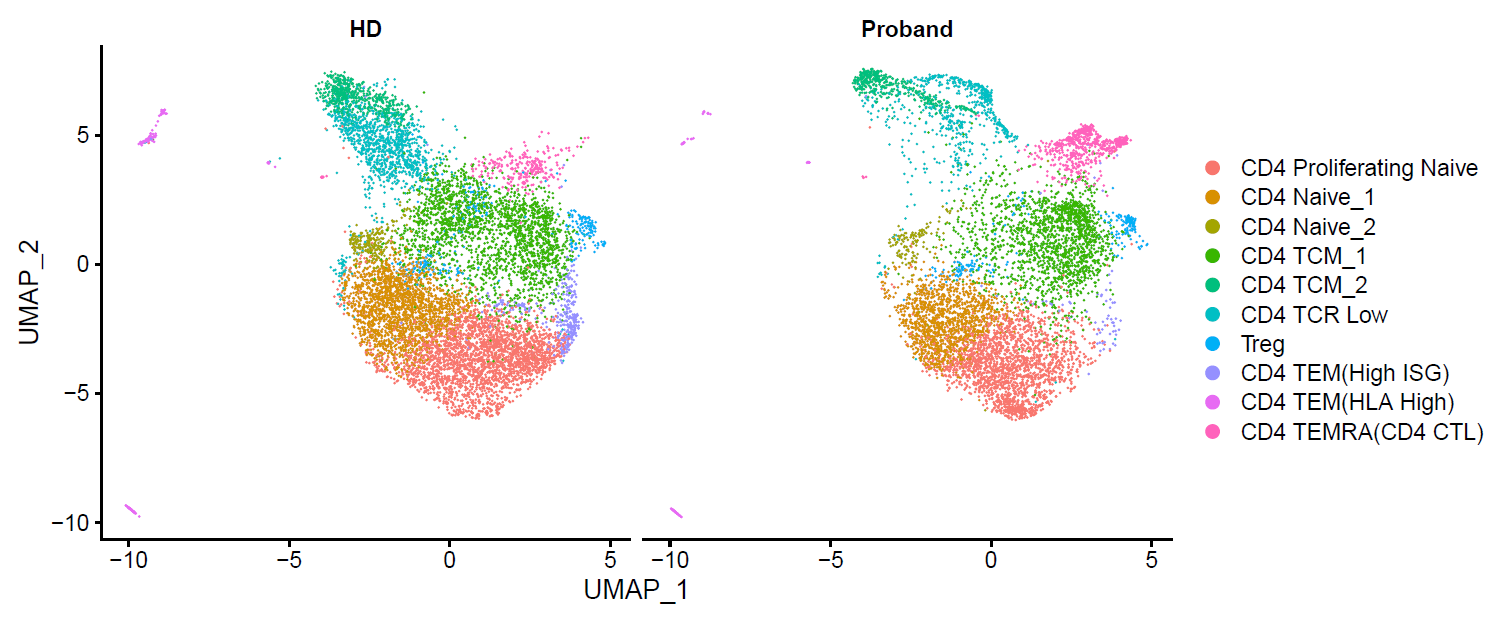

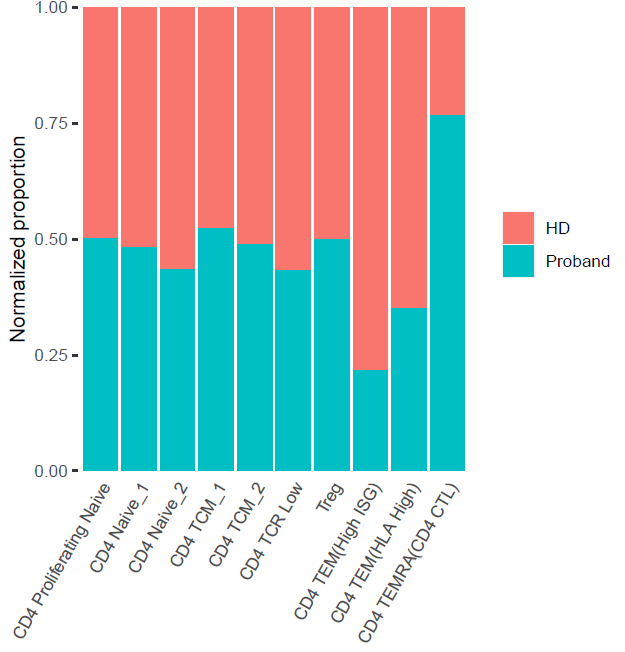


**A**


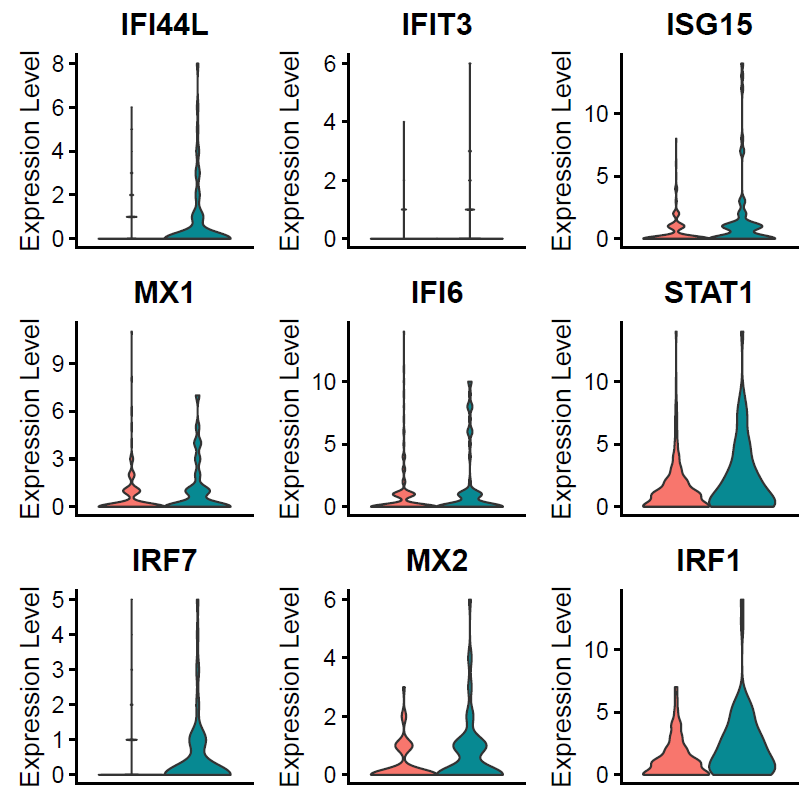

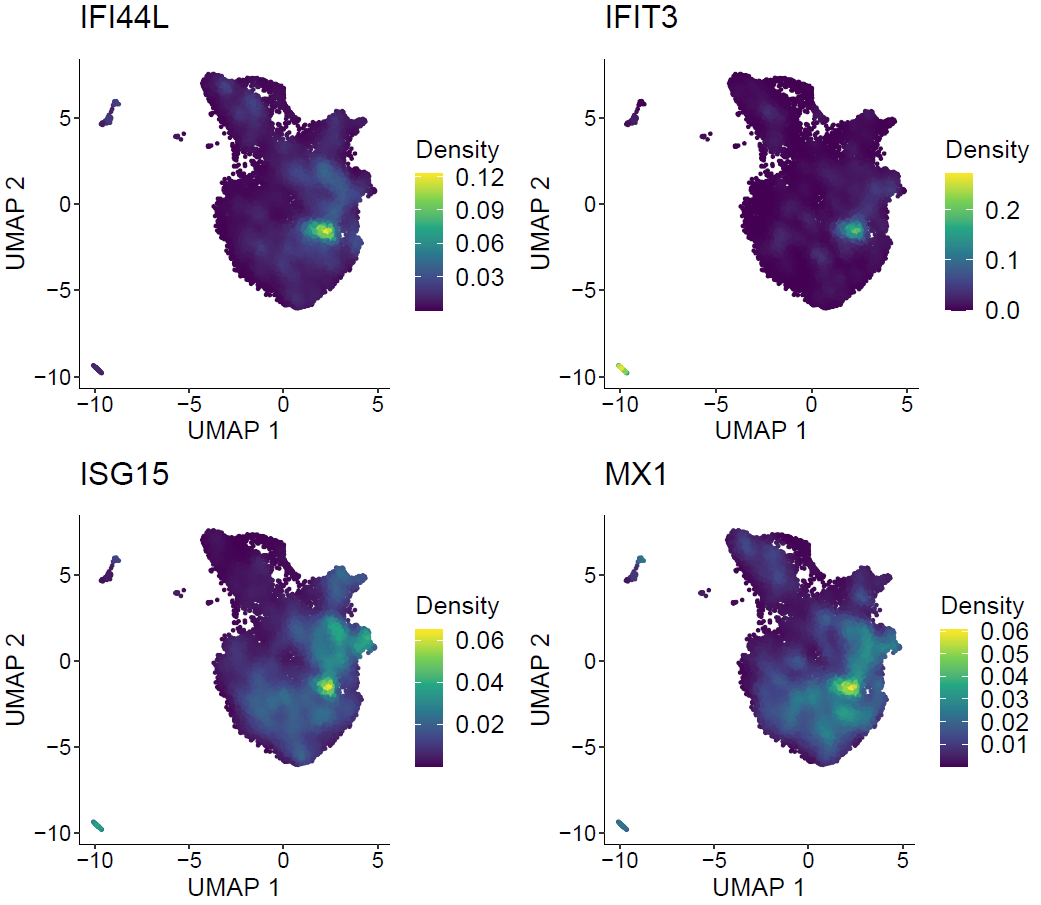


**C**

**B**

**D**

Supplementary Figure S3

(A) UMAP cluster divided by sample.

(B) Compositions of cell cluster by sample.

(C) Density plot produced by the Nebulosa package, showing the expression of High ISG related gene.

(D) Violin plots display the expression of High ISG signature genes between HD and Proband.
